# Body Mass Index and Perceived Labor Control: Could weight stigma explain differences in birth experience?

**DOI:** 10.21203/rs.3.rs-3142767/v1

**Published:** 2023-07-19

**Authors:** Anna R Whelan, Brock E Polnaszek, Olivia Recabo, Melissa A Clark, Adam K Lewkowtiz, Nina K Ayala

**Affiliations:** Women & Infants Hospital of Rhode Island, Alpert Medical School of Brown University; Women & Infants Hospital of Rhode Island, Alpert Medical School of Brown University; New York Medical College; Women & Infants Hospital of Rhode Island, Alpert Medical School of Brown University; Women & Infants Hospital of Rhode Island, Alpert Medical School of Brown University; Women & Infants Hospital of Rhode Island, Alpert Medical School of Brown University

**Keywords:** Labor agentry, control, patient experience, weight bias, weight stigma

## Abstract

**Background:**

Individuals with a body mass index (BMI) of ≥ 30 kg/m2 experience weight stigma when interacting with the healthcare system. There is limited data on how weight stigma impacts patient’s experience of obstetric care. This study aims to assess perceived control over the birth process and compare patients with BMI ≥ 30 to those with BMI < 30.

**Methods:**

We performed a secondary analysis of a cross-sectional study of term patients. Postpartum, participants completed the Labour Agentry Scale (LAS), a validated tool to assess perceived control over labor/birth. Continuous LAS scores were compared between patients with BMI < 30 and BMI ≥ 30.

**Results:**

There was no difference in LAS between those with BMI ≥ 30 and BMI < 30. When stratified by World Health Organization (WHO) class of BMI, those with BMI ≥ 40 had a significantly lower LAS scores than those with BMI < 30 (147 vs. 163, p = 0.02), however, this finding was no longer significant after controlling for length of labor and cesarean birth.

**Conclusion:**

Only participants with the highest BMI experienced decreased control over labor, and this finding was no longer significant after controlling for mode of delivery and length of labor. Further research is necessary into how weight stigma influences birthing people’s experience.

## Background

Weight stigma is defined as the social rejection and systematic devaluation of individuals living in larger bodies that do not conform to societal norms of body weight or shape^1^. Weight stigma permeates the medical field across all specialties^1^. Prior research has demonstrated that providers spend less time, establish less rapport and engage in less health education with patients living in larger bodies^1, 2^. Additionally, the patient experience of weight stigma varies, and increases with increasing body mass index(BMI), particularly for those who have a BMI ≥ 30^3^. This stigma leads patients to experience psychological distress, avoid seeking care and ultimately have poor health outcomes including increased rates of diabetes and cardiovascular disease^1, 2^.

Although weight stigma has been demonstrated to impact many aspects of care and patient experience, the potential effect of weight stigma among birthing people is less well understood. To our knowledge, there have been no studies to date which specifically evaluate patient experience of control over labor based on their weight. This study aims to assess perceived control over the birth process and compare patients with BMI ≥ 30 to those with BMI < 30.

## Methods

We performed a secondary analysis of a cross-sectional survey study of patients admitted to the labor and delivery unit at a single academic medical center, during the months of June and July 2021^4^. This study was approved by the institutional review board (#1691795). Eligible participants were nulliparous, English-speaking, and had singleton, non-anomalous pregnancies at gestational age ≥ 37 weeks. Participants were ineligible if they were scheduled for cesarean delivery (CD) or had a contraindication to labor. In the parent study,

After obtaining written consent, participants filled out a questionnaire which included the Labour Agentry Scale (LAS), a validated 29-item instrument that assesses childbirth control^5^. Trained researchers then performed a detailed chart review. For this secondary analysis, the primary outcome was LAS score, and participants were stratified by body mass index (BMI) at the time of delivery admission – calculated by stated or measured weight kg divided by height in meters-squared). BMI was first dichotomized into two groups—participants with BMI ≥ 30 kg/m^2^ versus those with BMI < 30 kg/m^2^—and then into multiple groups, and BMI < 30 was compared to each WHO class of obesity (class I BMI 30-34.9, class II BMI 35-39.9, class III BMI ≥ 40)^6^.

Data were analyzed using STATA v.15 (College Station, TX). Fisher’s exact test was performed for categorical variables and Wilcoxon Rank-sum for continuous variables. The primary outcome of score on the LAS was compared between those BMI < 30 and those with BMI ≥ 30 as well as those without obesity to those with WHO class of obesity as well (class I BMI 30-34.9, class II BMI 35-39.9, class III BMI ≥ 40) compared with BMI < 30 kg/m^2^. Multiple linear regression was performed controlling for a priori confounders of mode of delivery and labor length.

## Results

Of 149 participants in the original study, 87 (58.4%) reported a BMI ≥ 30. There were no differences in maternal age at delivery or participant stated race/ethnicity with the majority of participants identifying as white between those with a BMI ≥ 30 and those with BMI < 30 (Table 1). There were also no differences in the proportion of patients with medical or psychiatric comorbidities between those with BMI ≥ 30 and those with BMI < 30.

The majority of participants underwent spontaneous vaginal delivery (75.4% for patients with BMI < 30, 59.8% for patients with BMI ≥ 30) and there were no differences in mode of delivery between groups. However, those with BMI ≥ had significantly longer median labor time from admission to delivery than those with BMI < 30 (15 hours vs. 19 hours, p < 0.02). There was no difference in rate of NICU admission or need for neonatal therapy between groups (Table 1).

**Figure 1:**
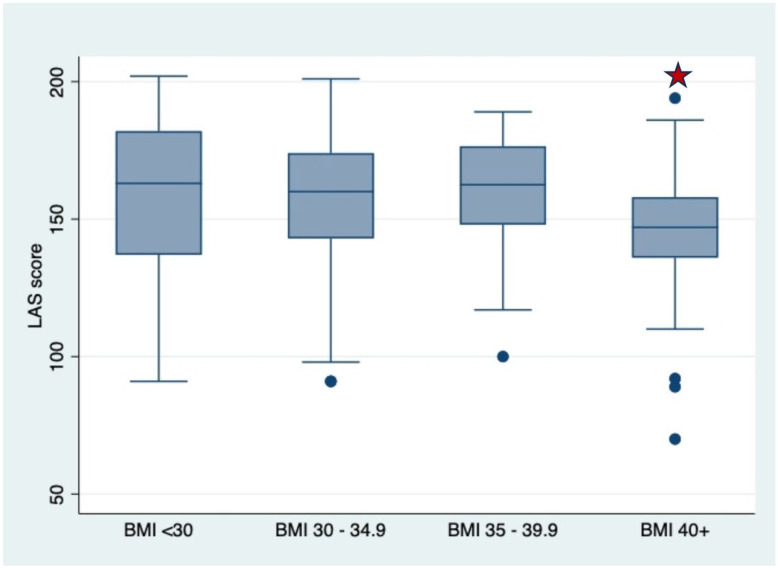
LAS Score by WHO BMI Class. Star indicates significance at p<0.05 when compared to BMI <30.

There were no significant differences in scores on the LAS for those who had BMI < 30 compared with BMI ≥ 30 (163 vs 154, p = 0.11). When analyzed by BMI category with BMI < 30 as the reference, those with BMI ≥ 40 had significantly lower LAS scores (147,vs 163, p < 0.02) (Fig. 1). However, after controlling for mode of delivery and labor duration, BMI was no longer significantly associated with differences in LAS score (Table 2).

## Discussion

In this study, there was no association of LAS score between those with BMI < 30 and those with BMI ≥ 30. For those with the highest BMI (≥ 40), lower scores on the LAS compared to those with BMI < 30 could be accounted for by the impact of cesarean delivery.

Contrary to prior literature, we did not detect a difference in mode of delivery among those with BMI ≥ 30 compared to patients with BMI < 30^7-9^. This may be a type II error due to our small sample size. As cesarean delivery could potentially explain the difference in LAS scores between patients with BMI > 40 compared to BMI < 30 a larger study is needed to confirm these findings. Additionally, interventions to decrease the rate of cesarean delivery, particularly among parturients with the highest BMIs could lead to improved patient experience.

To our knowledge this is the first study to assess perceived control over labor based on BMI as measurement of weight stigma. Weight stigma has been previously studied in primary care and adult medicine fields; however, data is lacking in obstetrics. Qualitative studies performed in the perinatal population in the UK and Australia have reported that patients experience negative attitudes and demeaning comments from providers and staff in the antepartum, intrapartum and postpartum period^10^, and report feeling like they are being “blamed” for the fetal risks of elevated BMI in pregnancy^10-12^. All of this culminates in patients reporting that the concern over their weight took the joy from their pregnancy^10, 12^.

Our study was limited by small sample size, which may increase the likelihood of type II error when analyzing differences between study groups. BMI was also calculated from patient stated weight and height on admission to labor and delivery, which may have led to measurement error as patients regularly underestimate/underreport weight^13^. Nevertheless, this study has many strengths. To our knowledge, this if the first study to assess patient experience of control over labor and how that interacts with patient BMI. The detailed chart review allowed us to assess and control for differences between those with an elevated BMI and those without elevated BMI.

Future qualitative studies are planned to assess both patient experience of weight stigma in pregnancy and provider attitudes toward those living in larger bodies with the ultimate goal of creating more inclusive obstetric care models.

## Conclusion

Patients with a BMI > 40 kg/m2 experienced less control over their labor, however this finding was no longer seen after controlling for cesarean delivery and length of labor. Future qualitative studies on obstetric patient experience of weight stigma and how it effects their labor is necessary.

## Figures and Tables

**Table 1:** Demographic and Delivery Characteristics Among Pregnant People With Body Mass Index < 30 Compared with ≥ 30 kg/m2

	BMI < 30 (N = 61)	BMI ≥ 30 (N = 87)	P-value
**Maternal Age, median (IQR)**	30 (26–33)	28 (24–31)	0.09
**Patient-reported race/ethnicity**	2 (3.3)	6 (6.9)	0.62
**Black**	8 (13.1)	18 (20.7)	
**Latina**	2 (3.3)	3 (3.4)	
**American Indian/Indigenous**	1 (1.6)	1 (1.2)	
**Asian/Pacific Islander White**	48 (78.7)	59 (67.8)	
**Indication for admission**	39 (63.9)	43 (49.4)	0.15
**Labor**	17 (27.9)	29 (33.3)	
**IOL (scheduled)**	5 (8.2)	15 (17.3)	
**IOL (unplanned)**			
**Length of labor (hours), median (IQR)**	15 (9–21)	19 (12–31)	**0.02**
**Mode of delivery** *	46 (75.4)	52 (59.8)	0.07
**SVD**	4 (6.6)	4 (4.6)	
**OVD**	11 (18.0)	31 (35.6)	
**CD**			
**NICU admission**	6 (9.8)	7 (8.2)	0.77
**Neonatal therapy** **	7 (11.5)	13 (14.9.9)	0.63

Data are N(%) unless otherwise stated. Significance at p < 0.05.

Fisher’s exact and Wilcoxon Ranksum tests used for analysis.

IQR = interquartile range, IOL = induction of labor, SVD = spontaneous vaginal delivery, OVD = operative vaginal delivery, CD = cesarean delivery.

* OVD consists of both forceps-assisted and vacuum-assisted deliveries.

** Neonatal therapy includes the need for supplemental O2, phototherapy for jaundice, neonatal antibiotics

**Table 2:** Labour Agentry Scale Scores by Obesity Status, Obesity Class and Multivariable Linear Regression

	Non-obese BMI < 30 (N = 61)			Obese BMI ≥ 30 (N = 87)		P-value	
Total LAS Median (IQR)	163 (137–182)			154 (141–174)		0.11	
By Class of Obesity							
	Non-obese BMI < 30 (reference) N = 61)	Class I BMI 30- 34.9 (N = 37)	P- value	Class II BMI 35-39.9 (N = 20)	P- value	Class III BMI > 40 (N = 30)	P-value
Total LAS Median (IQR)	163 (137–182)	163 (143–174)	0.34	162.5 (148–176.5)	0.45	147 (136–158)	**0.02**
Multivariable Linear Regression							
	Coeff (SD)			t			95%CI
Constant	166.43 (3.81)						
BMI > 40	−2.45 (4.39)			−0.56			−11.12,6.23
Cesarean delivery	−16.13 (4.85)			−3.32			**−25.71,−6.45**
Duration of labor (hrs)	−0.22 (0.12)			−1.84			−0.45,0.02

Significance at p < 0.05.

Wilcoxon Ranksum test used for analysis of LAS score between non-obese and obesity participants as well as between categories of obesity and non-obese.
